# Economic damage from natural hazards and internal migration in the United States

**DOI:** 10.1007/s11069-024-06987-2

**Published:** 2024-11-06

**Authors:** Marijn J. Ton, Hans de Moel, Jens A. de Bruijn, Lena Reimann, Wouter J. W. Botzen, Jeroen C. J. H. Aerts

**Affiliations:** 1 Institute for Environmental Studies (IVM), Vrije Universiteit Amsterdam, De Boelelaan 1087, 1081 HV Amsterdam, The Netherlands; 2 International Institute for Applied Systems Analysis (IIASA), Laxenburg, Austria; 3 Utrecht University School of Economics (U.S.E.), Utrecht University, 3508 TC Utrecht, The Netherlands

**Keywords:** Natural hazards, Migration, Gravity model, United States

## Abstract

In this paper, we analyze the impact of natural hazards on internal migration in the United States (US). The analysis uses Internal Revenue Service (IRS) tax records and analyzes different versions of a gravity model to assess the impact of economic damage caused by natural hazards on migration flows between counties. We find that natural hazards in the origin county are associated to larger outflows, with hurricanes exhibiting the most substantial impact on migration, followed by floodings and severe storms. These results emphasize the need for migration research that distinguishes between various types of hazards. Furthermore, we extend the gravity model to investigate whether people tend to relocate to relatively nearby areas in response to natural hazards, an established phenomenon in existing literature. For floods and severe storms, the analysis demonstrates that migration flows to nearby counties are comparatively larger than to distant counties. These findings may be concerning, as it implies that individuals could still be at a high risk of experiencing future disasters. Additionally, we provide several robustness checks to investigate to what extent our results are driven by extreme events in the dataset.

**Supplementary Information:**

The online version contains supplementary material available at 10.1007/s11069-024-06987-2.

## Introduction

As early as 1990, the IPCC reported that the single largest impact of climate change might be on human migration (IPCC and WMO 1992). A key issue is how climate change may increase the intensity, frequency, and duration of extreme weather events, such as floods, droughts and tropical cyclones. These weather extremes act as a direct driver of migration and displacement when people’s homes or livelihoods are suddenly destroyed, but also as an indirect driver through the gradual deterioration of economic and social conditions (Black et al. 2011; Rigaud et al. 2018).

In the United States (US), there has been an upward-moving trend in the annual number of natural hazards with more than one billion US Dollars in damages, which is not only due to climate change, but also due to increased exposure of vulnerable populations (Iglesias et al. 2021; Smith 2020). One of the most well-known disasters is Hurricane Katrina in 2005, which led to around 1.5 million people being displaced in the US (Groen and Polivka 2010). Displacements after an extreme event are usually temporary and over short distances, as many people return home soon after an event (McLeman and Gemenne 2018). Yet, after Katrina, temporary migration turned into permanent migration for many people (Fussell 2015).

Although the effects of Hurricane Katrina on migration are clear, statistical studies on migration in the U.S., which include multiple natural hazards, do not give a clear picture. For example, Boustan et al. (2020) find that natural hazards that caused more than 25 fatalities have led to 1.5% higher net out-migration in affected U.S. counties. Schultz and Elliott (2013), on the other hand, find that economic damage caused by disasters leads to a relatively higher population growth, which serves as a proxy for net in-migration. Strobl (2011) finds that impacts from hurricanes that make landfall increase both out-migration and in-migration rates, whereas Ouattara and Strobl (2014) show that hurricanes lead to higher out-migration rates only. Additionally, Fussell et al. (2017) find that hurricane damages barely impact future population growth in most U.S. counties, except for counties with a high past population growth and a high population density. For these counties, which make up about 2% of the entire sample of counties, yearly economic damages caused by hurricanes lead to lower population growth (indicating net out-migration). In contrast, 10-year cumulative economic damages lead to higher population growth (indicating net in-migration) (Fussell et al. (2017). Lastly, Winkler and Rouleau (2021) find that the impact of wildfires on migration in the US is low, but statistically significant.

Existing studies that use a statistical framework to analyze natural hazard induced migration in the US primarily employ models that are limited to estimations of (net) in- or outmigration *rates* or population changes (see studies mentioned above), which restricts their broader applicability. Instead, to investigate simultaneously where people originate from and where they move to, we need to analyze migration flows. To do so, a different model is required, such as a gravity model (Piguet 2022). In the traditional gravity model of migration, flows between two regions depend on their population sizes and the distance between them. Due to the limited availability of data on internal migration flows within a country, the gravity model has mainly been used to estimate the impact of climate change on international migration flows (see: Beine and Parsons 2015; Gröschl and Steinwachs 2017; Coniglio and Pesce 2015). However, migration in response to climate change is more likely to occur within nation’s borders than across borders (IPCC 2022). Nevertheless, several studies have analyzed migration flows between counties in the US, but these studies did not use the gravity model in a statistical modeling framework. For example, Hauer (2017) use county-to-county migration flows in a migration systems approach to calculate how the population distribution in the US changes due to sea-level rise-induced migration. Other examples are studies by DeWaard et al. (2016) and Curtis et al. (2015), which use county-to-county flows to study recovery migration after Hurricane Katrina applying a migration system approach.

In this paper, we study the impact of economic damage caused by natural hazards on migration flows in the US using statistical modelling techniques. We differentiate between several types of events: hurricanes, floods, severe storms, tornadoes, winter storms and earthquakes to analyze whether the type of hazard has a different impact on migration. In addition, we are interested in how far people move after a disaster. In the traditional gravity model, distance is one of the explanatory variables, but we add an additional variable where the distance interacts with the occurrence of a natural hazard. This allows us to analyze whether people tend to move to relatively nearby or distant counties in case of a natural hazard. In the literature, there is consensus that migration after natural hazards is usually short-term and over short distances (McLeman and Gemenne 2018), but we noticed that the studies in McLeman and Gemenne (2018) have mostly focused on developing countries or on a single event in a developed country, such as Hurricane Katrina. In this study, we estimate a panel regression based on the gravity model to statistically analyze if and to which degree this phenomenon is also visible for other events and other type of hazards in the United States.

The remainder of this paper is organized as follows. Section 2 outlines the methodology, starting with a description of the data and followed by an explanation of the gravity model and estimation technique. In Sect. 3, we present the results of the different gravity models, with an extensive robustness analysis provided in 3.2. Finally, Sect. 4 concludes with the Conclusions and Discussion.

## Methodology

In this section, we first describe the data used and then we explain how we apply the gravity model to estimate the impact of natural hazards on internal migration.

### Internal revenue service (IRS) data

Yearly county-to-county migration flows are obtained from IRS tax records, which cover around 87% of US households. Although certain demographic groups, such as students and older people, are underrepresented in the data because they typically do not file taxes, the IRS data still form the most comprehensive data set of migration flows in the US (Molloy et al. 2011; Winkler and Rouleau 2021). The data are available from 1990 onwards, but there are concerns about the quality of the data after 2010 and we, therefore, follow the advice of DeWaard et al. (2022) to only use IRS data from before 2011. One issue is that the IRS changed their data collection methodology after 2011, so the migration data are not directly comparable (DeWaard et al. 2022). For privacy reasons, the destination of migration flows smaller than 10 people is not provided in the data set, and only the total number of people in the small flows is provided. This means that for counties with a low population, the destination of migrants is relatively often unknown and we, therefore, remove counties that have a population lower than 5000, which leaves 2809 counties and reduces the dataset by 4.30%. In Table 1, we provide descriptive statistics of the migration flow data. The flows are measured in number of people and the flow rate is measured in percentages. The flow rate is defined as the flow divided by the county’s population in the previous year and multiplied by 100. We use the flow rate in our regression models so that we do not have to control for population growth.

**Table 1:** Descriptive statistics of migration flow data

	Observations	Min	25% percentile	Median	Mean	75% percentile	Max	Std
Flows	1,783,457	0	0	20	70.93	45	51,442	453.02
Flow rate (%)	1,783,457	0	0	0.0032	0.0545	0.0225	9.1599	0.1852

### Federal emergency management agency (FEMA) disaster damage data

In this paper, data from the FEMA are used to represent natural hazard losses. We have also checked other databases, such as SHELDUS hazard database, but we find that FEMA offers a more detailed spatial distribution of hazard damage.^1^

FEMA offers *public assistance* to states and counties in declared disaster areas to aid in the restoration of infrastructure, debris removal, and protective measures. Additionally, FEMA also provides *individual assistance* to households with damage to their homes, personal properties, vehicles, and more. However, this individual assistance is only provided to households not insured by the National Flood Insurance Program (NFIP). As a result, the extent of economic damage caused by natural disasters is influenced by insurance penetration rates, for which we lack yearly data. Moreover, the insurance penetration in the US is highly dependent on purchase requirements that only apply to the mapped 1 in 100 year flood zone, and not to other flood zones. Furthermore, Raker (2023) has demonstrated that the amount of disaster aid a household receives is correlated to their socio-economic and racial characteristics, with aid being directed towards higher-income households in whiter, more affluent communities. Therefore, we believe that the public assistance data give a more reliable representation of the economic damage resulting from natural hazards than individual public assistance data. Besides, the analysis can be extended by four years as public assistance data at the county level are available from 1999 onwards, while individual assistance data are available from 2003 onwards. However, Domingue and Emrich (2019) found that indicators of social vulnerability in a county are also correlated with the amount of public assistance a county receives, although these indicators were not consistent over the years studied in their paper. Also, it is important to emphasize that we use FEMA inspected (observed) damage data rather than FEMA subsidies, aiming to minimize potential biases caused by political and institutional processes. Lastly, there is some general critique about using ex-post damage data to assess the impacts from extreme events using global datasets, because economic damage exhibits a positive correlation with a country’s development level where damages may be reported more precisely (Felbermayr and Gröschl 2014). Nevertheless, because this study is focused on a single country with a homogenous administration of federal disaster assistance, we believe that this is a less important issue. Still, to investigate the effect of a potential correlation between hazard damage and income levels, we estimate an “income-corrected” model where we divide economic damage per capita by the income level in the county.

The FEMA differentiates between various types of disasters and provides a short description of each disaster (Table 2). In our time frame of 12 years, there are 707 disasters in total, where the disaster type severe storm is most present. Upon checking the description of the disasters, however, the description of severe storms is very similar to the descriptions of floodings and tornadoes in the database. For example, we find that the 2008 Midwest Floods are classified as severe storms, but we believe that it is more appropriate to classify them as floodings. Therefore, we re-categorize the severe storms based on a few simple rules. When the description of a disaster in the severe storms category contains Flooding, but not ‘Tornado’, then they are classified as ‘Flooding’. The number of floodings in the database thereby increases from 33 to 235. Similarly, when the description of a severe storms hazard contains ‘Tornado’, but not ‘Flooding’, then they are classified as ‘Tornado’, which increases the number of tornadoes from 14 to 45. Additionally, we assign tropical storms and typhoons to the hurricane category, which increases the number of hurricanes from 106 to 129. Also, there was one event classified as ‘Freezing’, which we added to the ‘Severe ice storm’ category. In Table 2, we provide some statistics of the disasters. It is noteworthy that hurricanes have the highest average damage per capita, as well as the highest maximum damage. The 99th percentile of damage per capita is provided because it is used in the robustness check.

**Table 2:** FEMA disaster types in the US over the period 1999–2010 (the original number of events per disaster type is given in brackets)

Disaster Type	Occurrence	Total number of counties hit	Average damage per capita	99th percentile damage per capita	Max damage per capita
Flood	235 (33)	2445	35.42	388.81	2510.42
Severe Storms	170 (419)	2923	31.55	323.46	4079.27
Hurricane	129 (106)	2305	82.70	1242.77	27,140.29
Tornado	45 (14)	336	27.65	310.49	1871.04
Snow	77 (77)	1047	3.68	15.91	70.20
Fire	16 (16)	330	9.57	24.96	2321.57
Earthquake	4 (4)	20	33.40	224.37	231.51
Severe Ice Storm	30 (29)	706	37.76	552.24	782.83

### Socio-economic data

Besides disaster damage, other variables that potentially affect migration are considered. Black et al. (2011) provide a conceptual framework for migration decisions and distinguish five drivers of migration: environmental, political, demographic, social, and economic drivers. We do not include all these drivers in the analysis, because, arguably, many of these drivers keep constant or change only modestly in a time span of 12 years. Hence, most drivers will be picked up by the fixed effects in the models that account for the effect of location on migration. Nevertheless, we include unemployment rates and income levels obtained from the U.S. Bureau of Labor Statistics (2021) and the U.S. Bureau of Economic Analysis (2021), respectively, because these two variables are usually included in gravity models (Coniglio and Pesce 2015; Mayda 2010). For seven counties that were heavily hit by hurricane Katrina, there were no unemployment data available for 2005 and 2006, so we replaced the missing data with the county’s mean unemployment rates.

### The gravity model

The gravity model is arguably the most popular model to explain migration dynamics (Poot et al. 2016). It is inspired by Newton’s law of gravity: Migration flows (${Flow}_{ij}$) are positively related to the population size in the origin and destination (${Pop}_{i}^{\beta }$ and ${Pop}_{j}^{\gamma }$), but negatively related to the distance between them (${Dis}_{ij}^{\delta }$), see Eq. 1 below. G is a scaling factor.

1 $$Flow_{ij} = G\frac{{Pop_{i}^{\beta } Pop_{j}^{\gamma } }}{{Dis_{ij}^{\delta } }}$$

The gravity model is grounded in microeconomic theory using random utility maximization (RUM) (Beine et al. 2016). The RUM model assumes that each individual evaluates all migration destination options based on their characteristics, such as population size and proximity. Based on this information, a person migrates to the destination that maximizes their utility under the condition that the utility in the destination is higher than the current utility in the origin. All the individual decisions can be aggregated into migration flows between regions. For an elaborative overview of the RUM model as a basis for the gravity model, we refer to Beine et al. (2016).

### The panel gravity model

In this paper, we employ a panel gravity model because we have data spanning several years. A key advantage of adopting a panel regression approach is that it helps to reduce the omitted variable bias by controlling for many (unobserved) factors. These factors include social networks, migration policies and specific amenities in regions. We control for these factors by including different combinations of fixed effects (here county-specific estimated constants).

In the panel gravity model, we include origin–destination fixed effects and time fixed effects. Origin–destination fixed effects are constants for each origin–destination county pair and they capture the fixed migration costs between pairs of counties (Maza et al. 2019; Royuela and Ordóñez 2018). In the traditional gravity model, distance is included as a proxy for the cost of migration from a given origin to a particular destination. However, the distance variable cannot capture all costs, because the cost of migration is also affected by other factors such as social networks and diaspora. When the distance variable is replaced by origin–destination fixed effects, i.e. constants for each combination of two counties, we include all time-invariant variables that affect migration between two counties, such as the distance between them, whether they share a border, existing diaspora, etc. The origin–destination fixed effects thereby capture the fixed (time-invariant) migration costs of moving between two specific counties. The time-fixed effects are constants per year and they capture yearly migration shocks common to all counties (Maza et al. 2019; Royuela and Ordóñez 2018). For example, when there is less migration in a given year at the national level, then the time fixed effects can capture this effect.

#### Base model: fixed migration costs

**Fig. 1:**
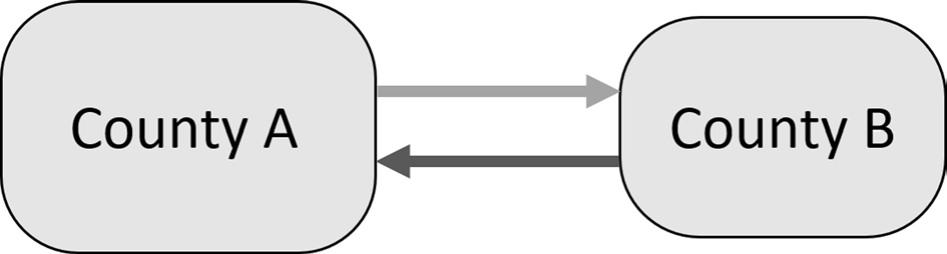
Base model: Fixed migration costs between county pairs

In the first model, we only include fixed effects and the two control variables. In Fig. 1, we schematically draw the gravity model (‘Base model’) where the migration flows, represented by the arrows, are solely based on origin–destination fixed effects. The two arrows have a different color, because the fixed migration costs and thus the size of the migration flows (rates) differ for each origin–destination pair.

Since origin–destination fixed effects in the *Base model* do not capture changes over time in the attractiveness of counties, we control for changes in economic conditions by including GDP and unemployment rates in the regression. We do not include other control variables because the time span of this study is limited (12 years) and we anticipate that there is only minimal variation over time in the attractiveness of counties due to changes in, for example, migration policies or institutions. Equation (2) provides the specification of the base model, where ${Flow}_{i,j,t}$ represents the flow rate from county *i* to county *j* in year *t*, ${\mu }_{i,j}$ represents the origin–destination fixed effects, ${\theta }_{t}$ is the time fixed effects and we include the two control variables.

2 $$Flow_{i,j,t} = exp\left( {\mu_{i,j} + \theta_{t} + \delta_{1} \log \left( {\frac{{GDP_{j,t} }}{{GDP_{i,t} }}} \right) + \delta_{2} \log \left( {\frac{{Unemploy_{j,t} }}{{Unemploy_{i,t} }}} \right)} \right)$$

#### Extended model: damage caused by natural hazards

**Fig. 2:**
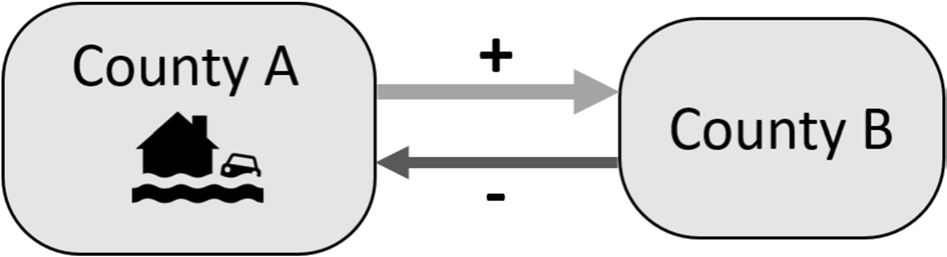
Extended model with damage per type of hazard

Next we add economic damage caused by natural hazards, resulting in the *Extended model*. This model is explained in Fig. 2, where county A is hit by a flood. The flood event increases the outflow to county B, indicated by ‘ + ’, and it decreases the flow from county B to county A, because county A has become less attractive as a destination.

In Eq. (3), we present the base model extended: ${\beta }_{h}$ captures the impact of damage in the origin county for each type of disaster *h* and ${\varphi }_{h}$ captures the impact of damage for different disaster types in the destination county.

3 $$Flow_{i,j,t} = exp\left( {\mu_{i,j} + \theta_{t} + \delta_{1} \log \left( {\frac{{GDP_{j,t} }}{{GDP_{i,t} }}} \right) + \delta_{2} \log \left( {\frac{{Unemploy_{j,t} }}{{Unemploy_{i,t} }}} \right) + \mathop \sum \limits_{h = 1}^{H} \left( {\beta_{h} Damage_{i,t}^{h} + \varphi_{h} Damage_{j,t}^{h} } \right){ }} \right)$$

#### Interaction model: including distance * hazard

**Fig. 3:**
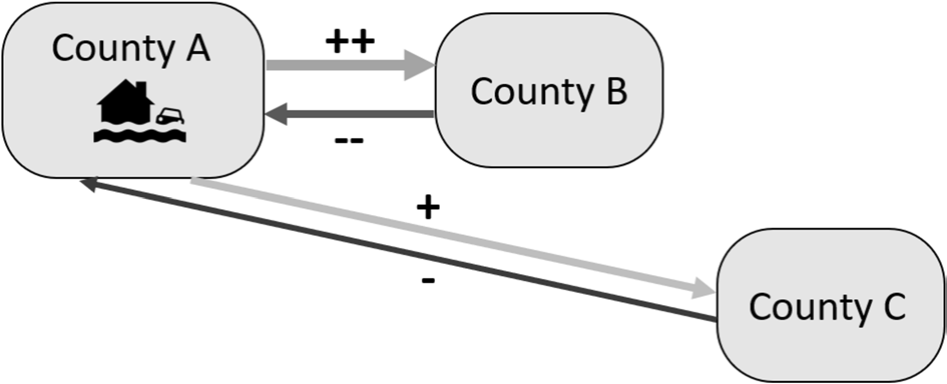
Interaction model with distance multiplied by hazard dummies

In the previous model in Fig. 2 and Eq. 3, we assume that all flows are affected by the same percentage in the event of a hazard *h*. In other words, when county A is hit by a flood, then the flows to all other counties are increased by ${\beta }_{h}$ (where *h* is flood). However, literature suggests that the migration response after a natural hazard is usually short-term and over short distances (McLeman and Gemenne 2018). To test whether this phenomenon also applies to disasters in the US, we introduce an interaction variable for each type of hazard in a new model (*Interaction model)* depicted in Fig. 3 and Eq. 4. The interaction variable is represented by a dummy variable *Occurrence* that is activated in the year *t* a hazard occurs in a county *i* or *j* and then multiplied by the distance (*Distance*_*i,j*_) between two counties.

In Fig. 3, we added a third county C, which is further away from county A than B. If the hypothesis is true, then we find that the increase in the flow from A to B in response to a flood in A is relatively larger than the increase in flow from A to C, indicated by the +  + and + , respectively. For symmetry concerns, we also include an interaction variable for the occurrence of hazards in the destination. It may be possible that people in nearby counties are more aware of the hazard damage and risk and consequently are less inclined to relocate to the disaster-hit county compared to people in distant counties. In this example, the reduction in the flow from counties B to A would be more substantial than the decrease in the flow from counties C to A, indicated by – and -, respectively. However, the literature does not provide examples of this effect.

The *interaction model* is given in Eq. (4) below. The coefficient ${\gamma }_{h}$ captures the interaction effect when a hazard occurs in the origin county *i* and ${\varphi }_{h}$ captures the interaction effect in the destination county *j*.

4 $$Flow_{i,j,t} = exp\left( {\mu_{i,j} + \theta_{t} + \delta_{1} \log \left( {\frac{{GDP_{j,t} }}{{GDP_{i,t} }}} \right) + \delta_{2} \log \left( {\frac{{Unemploy_{j,t} }}{{Unemploy_{i,t} }}} \right) + \mathop \sum \limits_{h = 1}^{H} \left( {\beta_{h} Damage_{i,t}^{h} + \varphi_{h} Damage_{j,t}^{h} + \gamma_{h} \left( {Occurrence_{i,t}^{h} *Distance_{i,j} } \right) + \varphi_{h} \left( {Occurrence_{j,t}^{h} *Distance_{i,j} } \right)} \right){ }} \right)$$

### Estimation method

Following Coniglio and Pesce (2015), Gröschl and Steinwachs (2017) and Beine and Parsons (2015), we adopt the Poisson Pseudo-Maximum Likelihood (PPML) method to estimate the panel gravity models. The advantage of PPML compared to Least Squares estimation is that it is consistent under heteroscedasticity (i.e., the variance of the residuals are not assumed to be constant across different values of the independent variables) and it allows for zero values in the flows between regions. However, in our data set, nearly 99% of the county pairs show no migration in the entire time span of 12 years. This severe imbalance would impose significant challenges in estimating the models, and we therefore choose to exclude these county pairs from the data set. This would normally introduce an estimation bias, since the county pairs lacking migration are not randomly distributed. However, this bias is solved by including the origin–destination fixed effects. For county pairs with no migration between them, the origin–destination fixed effects can go towards negative infinity, rendering the other variables in the regression irrelevant in explaining the lack of migration between these counties. In other words, we assume that the migration costs of moving between these counties is too high to ever become beneficial, even in the event of natural hazards or income shocks. When we do not include origin–destination fixed effects in the panel gravity model, we cannot rely on this assumption, and the coefficients could potentially be biased by excluding the county pairs lacking migration. This provides further support for incorporating origin–destination and time fixed effects into the model.

## Results

In the next section, we present the results of the different gravity models. Additionally, we provide an extensive robustness check, where we distinguish between extreme events and less severe events to assess whether our results driven by extreme events in the dataset. In Sect. 3.3, we give an illustrative example to make our results more concrete and accessible.

### Gravity model

Table 3 provides the results of the different gravity models. The base model (1) (Fig. 1) only contains origin–destination fixed effects, time fixed effects and the income and unemployment differentials. In the extended model (2) (Fig. 2), damage variables are added, and the *Interaction model* (3) (Fig. 3) includes the interaction variables.

**Table 3:** Results of the three different gravity models: (1) Base model, (2) Extended model, (3) Interaction model

	Base model (1)	Extended model (2)	Interaction model (3)	
Income differential	0.1250***	0.0873***	0.0876***	
	(0.0161)	(0.0127)	(0.0127)	
Unemployment differential	-0.8910***	-0.896***	-0.0893***	
	(0.0041)	(0.0040)	(0.0040)	
		Economic damage	Economic damage	Interaction variable
O_hurricane		0.0933***	0.0932***	0.0080*
		(0.0069)	(0.0069)	(0.0045)
D_hurricane		-0.1037***	-0.1039***	0.0171***
		(0.0149)	(0.0149)	(0.0050)
O_storm		0.0535***	0.0552***	-0.0195***
		(0.0165)	(0.0166)	(0.0069)
D_storm		0.0135	0.0133	0.0133*
		(0.0107)	(0.0107)	(0.0080)
O_flood		0.0721***	0.0751***	-0.0129***
		(0.0240)	(0.0240)	(0.0046)
D_flood		-0.1411***	-0.1336***	-0.0205***
		(0.0401)	(0.0397)	(0.0046)
O_tornado		0.0644	0.0617	0.0165
		(0.0484)	(0.0486)	(0.0145)
D_tornado		-0.0047	-0.0055	0.0283*
		(0.0881)	(0.0882)	(0.0153)
O_fire		-0.0084	-0.0063	-0.0134
		(0.0084)	(0.0086)	(0.0095)
D_fire		-0.0604***	-0.0589***	-0.0048
		(0.0093)	(0.0093)	(0.0054)
O_snow		1.0845*	0.9820	0.0091**
		(0.6240)	(0.6596)	(0.0045)
D_snow		-1.5585**	-1.7902***	0.0192***
		(0.6460)	(0.6792)	(0.0062)
O_icestorm		0.0508	0.0424	0.0206
		(0.0653)	(0.0656)	(0.0126)
D_icestorm		-0.1163	-0.1251*	0.0363*
		(0.0708)	(0.0712)	(0.0191)
O_earthquake		0.2125	0.2616	-0.0288*
		(0.2508)	(0.2502)	(0.0172)
D_earthquake		-0.8313**	-0.5560*	-0.0674***
		(0.3477)	(0.3173)	(0.0217)
Pseudo R2	0.4663	0.4666	0.4666	

*Note: O denotes the variables in the origin county and D denotes the variables in the destination county*

In model (1), we see that the income differential (i.e. income in the destination divided by income in the origin) has a positive impact on migration flows. This means that a decrease in income in the origin county or increase in income in the destination is associated to larger migration outflows. More specifically, when income in the origin county increases (decreases) by 1%, this relates to a decrease (increase) in the outward flow rate of 0.125%. However, when we include damage in model (2), this effect decreases from 0.1250 to 0.0873. The reduction in the coefficient estimate suggests that natural hazards capture part of the variation in the flow rate that was previously attributed to income. This implies that the impact of income on migration would be overestimated if natural hazards are not taken into account, highlighting the importance of including natural hazards to obtain a more accurate estimation.

Given the growing interest of researchers in exploring the indirect impact of environmental factors on migration via, for example, economic drivers, such as employment opportunities and income (Black et al. 2011), we want to analyze the impact of income and unemployment rates on migration further. To investigate the indirect impact of natural hazards on migration through income and unemployment rates we follow Beine & Parsons (2015) and we regress the economic damage caused by natural hazards on the unemployment differential and income differential. For the estimated coefficients we refer to the Supplementary Information, Table S1, but we highlight some of the results. We find, for example, that a hurricane with $1000 dollar per capita damage is associated with a 1.11% increase in the income differential and a 1.53% decrease in the unemployment differential. From the Base model (1), we saw that a 1% increase in the income differential relates to a 0.13% increase in the outward flow rate and a 1% decrease in the unemployment differential relates to a 0.09% increase in the outward flow rate. Combining these numbers, this suggests that a $1,000 dollar hurricane increases the outward flow rate by 0.14% through income and by 0.14% through unemployment. Hence, the indirect impact of hurricanes on migration through income and the unemployment rates is almost negligible.

Furthermore, since Felbermayr and Gröschl (2014) observed a correlation between hazard damages and a country’s GDP levels, we investigated whether our results are affected by a potential correlation between economic damage from hazards and a county’s income level. For example, counties with a lower income level might experience lower damages because buildings are less valuable. However, we find only minor changes in the coefficient estimates between the income-corrected model and the original model, with no clear indication of over- or underestimation of the coefficients. The detailed results are given in the Supplementary Information, Table S2.

Compared to the indirect impact of hazards on migration, the estimated coefficients of the Extended model (2) suggest that the direct impact of economic damage from hurricanes on migration outflows is much larger. The coefficients of the hazard damage can be interpreted as follows: $$\left({e}^{\beta *c}-1\right)*100\%$$, where *β* is the estimated coefficient and *c* is the damage caused by the hazard. When *c* is $1,000 damage caused by hurricanes in the origin county, it results in a $$\left({e}^{0.0933*1}-1\right)*100\%=9.78\%$$ increase in the (outward) flow rate. For other hazards we find similar results: A $1,000 dollar per capita damage caused by storms or floods in the origin county leads to 5.50% and 7.48% increase in outflows, respectively. The impact of snow storms on migration is substantially larger than the impact of the other hazards, but this can be explained by the low economic damage caused by snow storms. On average, snow storms cause damage amounting to 3.68 US dollars, whereas hurricanes or floods result in much higher damage, averaging at 82.70 and 35.42, respectively (see Table 2).

Besides the considerable impact of natural hazards on out-migration, the estimated coefficients also suggest that natural hazards can have a significant impact on in-migration. Except for storms, tornadoes, and ice storms, natural hazards in the destination are correlated to lower migration inflows. Again, especially the impact of hurricanes is large on migration inflows, but also the impact of floodings is substantial. For example, a 1,000 US dollar per capita damage caused by hurricanes and floods is associated with a decrease in the inward flow rate of respectively 9.85% and 13.16%.^2^ The decrease in migration flows to the destination (in-migration flows) could be due to the reduced attractiveness of the county after a hazard. On the other hand, Pais and Elliott (2008) suggests that disasters could act as pull factor attracting migrants due to new economic opportunities through an influx of federal and private insurance money. Our results, however, do not support this hypothesis and this could be attributed to our focus on the immediate impact of disasters on migration rather than the cumulative effect of disasters over multiple years. This align with the results of Fussell et al. (2017) who found that within a one-year timeframe, hurricane events decrease population growth, whereas hurricane events accumulated over 10 years increase population growth (in counties with a growing and high-density population).

In the final model (3), we incorporated interaction variables for each type of hazard where we multiplied the distance between two counties with a dummy variable that becomes 1 when a hazard occurs in a county and is 0 otherwise. In the first column of model (3), the coefficients of the economic damage are reported and in the second column of model (3) the coefficients of the interaction variable are displayed. For storms and floods in the origin (affecting outward migration), we find a negative coefficient significant against a 1% level, which means that migration flows to more nearby counties are relatively large as compared to flows to more distant counties. For example, a storm (flood) of 1,000 dollar damage per capita increases the outward flow rate by 5.57% (7.73%) when two counties are 50 miles apart and 4.65% (7.11%) when the counties are 500 miles apart.^3^ Hence, the flows to nearby counties are comparatively larger than to distant counties –although the difference is small. However, the interaction variables of hurricanes and snow storms is positive, suggesting that flows to distant counties are comparatively larger than flows to nearby counties. We delve deeper into these mixed results in Sect. 3.2. In addition, it is noteworthy that the R2 of the extended model (model 2) and the interaction model (model 3) are identical. This suggests that the interaction variables have a minor contribution to the explanatory power of the model, even though being significant.

Furthermore, the interaction variables for hazards in the destinations give mixed results. In case of floodings and earthquakes, the inward flows from nearby counties are relatively large as compared to inward flows from distant counties, while for hurricanes, storms, tornadoes, snow storms and ice storms we find the opposite. It should be noted, however, that the interaction variable of tornadoes, snow storms and ice storms is only significant against a 10%. One reason why flows from distant counties may be comparatively larger than those from nearby counties is that people living in close proximity may be more aware of the county’s loss in attractiveness or risks associated with hazards than people living in distant counties. At the same time, the reasoning can be reversed: people in nearby counties may be more likely to relocate to the disaster-hit county and help in reconstruction work because they are more aware of the damage caused by the hazard. However, it is mostly undocumented immigrants from Central America that help in disaster reconstruction work (Brown et al. 2022) and they do not appear in the IRS tax records data. In this perspective and given that the majority of coefficients is positive, the first reasoning would be more plausible: people further away might be less aware of the hazard damage and associated disaster risks, so they still relocate to the disaster-hit county. However, there is lack of research on this matter and no supporting evidence in the literature.

### Robustness checks

Our dataset of natural hazards contains one event that was exceptionally destructive, Hurricane Katrina. To investigate to what extent our results are affected by extreme events, such as Hurricane Katrina, we conduct an additional analysis where we separate each hazard variable into two variables: one variable contains per capita economic damages until the 99th percentile and the second variable contains the “extreme” per capita economic damages starting from the 99th percentile. The values of the 99th percentile of each hazard are given in Table 2.

After creating the variables for extreme and less severe hazards, we can evaluate whether our results are driven by the extreme events by testing whether the coefficients of the less severe hazards are still significant. Also, we can test whether the difference in parameter estimates is significant. In Table 4, we present the coefficient estimates of the extended model where we distinguish between extreme events and less severe events, along with the estimates of the original extended model.

**Table 4:** Estimated coefficients of extended model with extreme events

	Original extended model	Extended model with extreme events		
		Less severe economic damage < 99th percentile	Extreme economic damage > = 99th percentile	Test statistic with p-value
O_hurricane	0.0933***	0.3659***	0.0976***	0.2682***
	(0.0069)	(0.0365)	(0.0057)	(0.0384)
D_hurricane	-0.1037***	-0.3656***	-0.1074***	-0.2582***
	(0.0149)	(0.0771)	(0.0151)	**(0.0836)**
O_storm	0.0535***	0.1200**	0.0371**	0.0829
	(0.0165)	(0.0547)	(0.0166)	(0.1635)
D_storm	0.0135	0.2530***	0.0010	0.2520
	(0.0107)	(0.0657)	(0.0114)	(0.0663)
O_flood	0.0721***	0.0965*	0.0738***	-0.0470
	(0.0240)	(0.0525)	(0.0267)	(0.0852)
D_flood	-0.1411***	-0.2756***	-0.0652	-0.2104***
	(0.0401)	(0.0684)	(0.0410)	**(0.0784)**
O_tornado	0.0644	0.2187*	0.0330	0.1856
	(0.0484)	(0.1291)	(0.0543)	(0.1400)
D_tornado	-0.0047	0.2808**	-0.0707	0.3515
	(0.0881)	(0.1421)	(0.1043)	(0.1763)
O_fire	-0.0084	-2.6039	-0.0069	-2.5970
	(0.0084)	(2.3408)	(0.0085)	(2.3407)
D_fire	-0.0604***	-1.9002**	-0.0589***	-1.8412**
	(0.0093)	(0.9246)	(0.0095)	(0.9245)
O_snow	1.0845*	2.2123***	-0.3753	2.5875**
	(0.6240)	(0.7736)	(0.9484)	(1.1743)
D_snow	-1.5585**	-2.3539***	-0.8557	-1.4982
	(0.6460)	(0.7784)	(1.1167)	(1.3169)
O_icestorm	0.0508	0.0736	0.0283	0.0453
	(0.0653)	(0.0973)	(0.0800)	(0.1203)
D_icestorm	-0.1163	-0.0677	0.1824*	0.1147
	(0.0708)	(0.0941)	(0.0993)	(0.1313)
O_earthquake	0.2125	-0.0481	0.5023***	-0.5504
	(0.2508)	(0.4653)	(0.1310)	(0.4835)
D_earthquake	-0.8313**	-2.0095**	-0.3310	-1.6784*
	(0.3477)	(0.8209)	(0.3056)	(0.8760)
Pseudo R2	0.4666	0.4666		

Overall, the coefficients of the original model seem to be more closely aligned with the coefficients of the extreme hazards than with those of the non-extreme hazards, especially when we consider hurricanes, severe storms, floods and wildfires. This suggests that the extreme events in the dataset have a considerable effect on the estimated coefficients. Nevertheless, the coefficients of less severe events did not become insignificant and in fact, the estimated coefficients are amplified in the case of hurricanes, storms, floods, tornadoes, wildfires and snow storms. Still, we want to emphasize that our study employs data on the economic damage caused by hazards. This means that while non-extreme events seem to have a larger relative impact compared to extreme events, the absolute impact of larger hazards (i.e., with higher economic damage) may still be larger than that of hazards below the 99th percentile. Given the apparent non-linear impact of hazards on both outward and inward migration flows, taking a logarithm of economic damage may be more suitable. By taking the logarithm, the extreme values in economic damage are reduced, which could result in a better model fit. In addition, it is worth examining whether the impact of hazards on migration remains significant after applying a logarithmic transformation to the data. In Table 5, we present the results of the log extended model and the log interaction model.

**Table 5:** Estimated coefficients of the log-transformed models

	Log extended model (2)	Log interaction model (3)	
Income differential	0.1258***	0.1257***	
	(0.0156)	(0.0156)	
Unemployment differential	− 0.0890***	-0.0888***	
	(0.0040)	(0.0040)	
	Economic damage	Economic damage	Interaction variable
O_hurricane	0.0320***	0.0344***	-0.0317***
	(0.0069)	(0.0036)	(0.0065)
D_hurricane	− 0.0109***	-0.0132***	0.0297***
	(0.0015)	(0.0017)	(0.0057)
O_storm	0.0044***	0.0053***	-0.0290***
	(0.011)	(0.0012)	(0.0072)
D_storm	0.0024**	0.0018	0.0085
	(0.0012)	(0.0013)	(0.0083)
O_flood	0.0031***	0.0035***	-0.0167***
	(0.0010)	(0.0011)	(0.0049)
D_flood	− 0.0044***	-0.0039***	-0.0164***
	(0.0012)	(0.0012)	(0.0050)
O_tornado	0.0085***	0.0085***	-0.0025
	(0.0025)	(0.0025)	(0.0165)
D_tornado	0.0048*	0.0051*	-0.0120
	(0.0027)	(0.0028)	(0.0188)
O_fire	− 0.0101**	-0.0086*	-0.0124
	(0.0046)	(0.0048)	(0.0105)
D_fire	− 0.0121***	-0.0146***	0.0118
	(0.0027)	(0.0031)	(0.0075)
O_snow	0.0060**	0.0058**	0.0050
	(0.0024)	(0.0027)	(0.0049)
D_snow	− 0.0215**	-0.0050*	0.0183***
	(0.0053)	(0.0027)	(0.0065)
O_icestorm	0.0023	0.0022	0.0156
	(0.0020)	(0.0021)	(0.0131)
D_icestorm	− 0.0028	-0.0037*	0.0480**
	(0.0020)	(0.0021)	(0.0195)
O_earthquake	0.0109	0.0135	-0.0469**
	(0.0082)	(0.0089)	(0.0196)
D_earthquake	− 0.0215***	-0.0127**	-0.0563**
	(0.0053)	(0.0059)	(0.0242)
Pseudo R2	0.4664	0.4664	

We cannot compare the sizes of the coefficients of the original extended model and the logarithmic extended model, but we do see that the signs of the coefficients remain the same. In addition, the p-values of the estimated coefficients are roughly similar, where the coefficients in the logarithmic model seem to be slightly more accurately estimated (i.e., relatively lower standard errors and thus higher *p*-values). However, the pseudo R2 of the logarithmic model is slightly lower than the R2 of the original model, suggesting that the original model provides a better fit to the data. We suspect that the log transformation is probably not the most effective way to deal with the non-linearities in the data. To explain further, we need to look into the interpretation of the coefficients of economic damage variables. For small changes in economic damage per capita, the change in flow rate is approximated by β%. However, for large changes in damage, the change in the flow rate is absolute. In Sect. 3.1, we calculated the estimated effect of a hurricane with a 1000 USD damage per capita, which corresponds to an increase of 100,000%.^4^ The absolute change in the flow rate is given by the following formula: exp(0.032*log(new damage)) – exp(0.032*log(old damage)) = 0.1008. Hence, for a mean flow rate of 0.0545%, the new flow rate is estimated to be 0.1553% after the event of a hurricane with 1000 USD damage per capita. When the initial flow rate was 1%, the flow rate would increase by 0.1008% as well. In other words, the estimated impact of economic damage on the flow rate is additive when we log transform economic damage per capita, rather than multiplicative, as is the case for the economic damage in levels in the original model. This could explain why the original model has a better performance.

Nevertheless, the logarithmic model provides an additional robustness check to our results. As we can see from Table 5, economic damages from hurricanes, storms, floods and tornadoes have a significant impact (*p*-level < 0.01) on migration outflows. Additionally, hurricanes, floods, fires and earthquakes are associated with a decrease in migration inflows. This aligns with the previous results for the original model without log-transformation. In addition, we find that the interaction effects of hurricanes, storms and floods on outward migration are significant against a 1% level and negative, suggesting that people tend to move over shorter distances in the event of these hazards. In the original model, we found that people tend to move over longer distances in the event of a hurricane (although only significant against a 10% level). We suspect that this effect was caused by the extreme hurricane event in the dataset, Hurricane Katrina. This is consistent prior research, such as Fussell et al. (2014) who demonstrated that people spread across the entire United States after Katrina. We believe that this effect prevailed in the original model, whereas in the logarithmic model, the effect of extreme events is dampened by the logarithmic transformation of the data, reducing the influence of outliers like Hurricane Katrina on the estimates.

### Illustrative example of the results

**Fig. 4:**
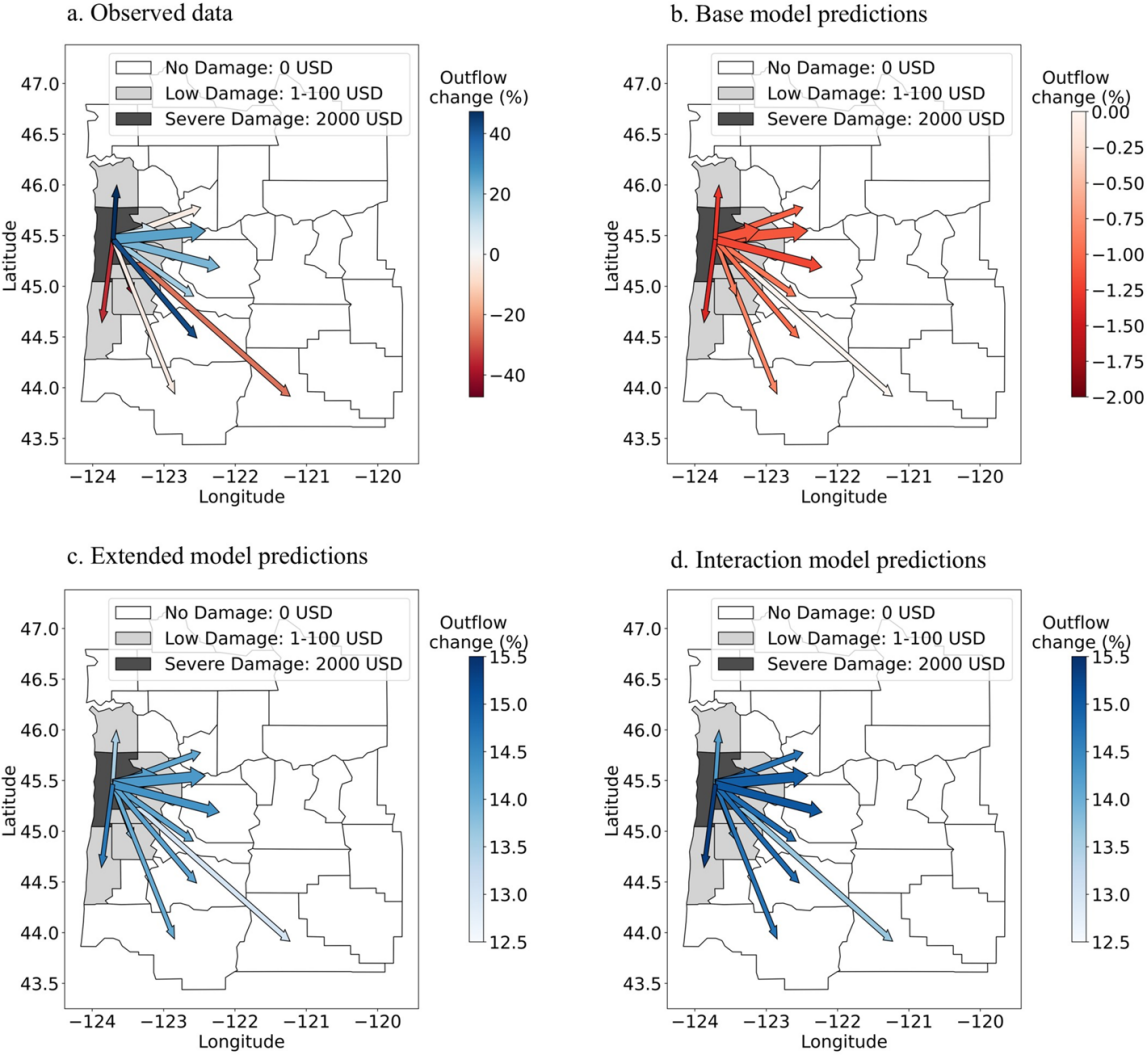
Change in migration outflow rate in Tillamook county after a severe flooding

To make our results more concrete and accessible, we provide a visual example of the change in migration flows in the event of a hazard. In Fig. 4, we depict the change in the migration outflow rate after a severe flooding in Oregon. The flooding occurred in 2007 as a result of coastal storms and was especially severe in Tillamook county, where the damage per capita reached 2000 USD. In Fig. 4, we plot the percentage change in outflows from Tillamook county between 2006 and 2007. Panel a) shows the observed change in the migration flow rate from 2006 to 2007. The data exhibit substantial variation, going from a 35% decrease to a 42% increase in the flow rate. As shown in panel b, c and d, the model predictions (base, extended and interaction model) cannot explain the variation in the flow rate. In panel b, the base model predicts a small decrease (on average -0.9%) in the outflows, which is due to a relative increase in income and relative decrease in the unemployment rate in Tillamook county. Contrary to the base model, the extended and interaction model (panel c and d) predict an increase in migration outflows, since these models take into account the impact of natural hazards. The interaction model predicts a slightly higher increase in the outflow rate, as the estimated coefficient is slightly higher compared to the extended model. In the interaction model, the flow rate decreases by the distance between counties in the event of a hazard. However, since the counties on the map are relatively close, this effect is not visible in the figure. On average, the extended model predicts an increase in the outflow rate of 14.1% and the interaction model predicts an increase in the outflow rate of 14.8%. This latter prediction is closer to the observed average increase of 20.8%.

## Discussion and conclusions

In this study, we analyze the impact of different types of natural hazards on migration flows in the US using a gravity model. The gravity model allows us to analyze the impact of natural hazards on inward and outward migration simultaneously. Our results are generally consistent. We find that natural hazards in the origin county are associated with larger outflows, and hurricanes, floodings and storms seem to have the largest effect.

- *Impact on outward migration*: Hurricanes, floodings and severe storms seem to have the largest impact on outward migration and these effects are significant at a 1% level. These findings are confirmed by our robustness check, which differentiates between extreme and less severe disasters. In fact, the relative impact of less severe hazards seems higher than the impact of extreme hazards. To address these apparent non-linear effects, we estimated a log-transformed model, which dampens the impact of extreme events on the model estimates. The log-transformed model further supports our results. However, the model fit (pseudo R2) is lower than that of the original model, which may be attributed to the difference in interpretation of the coefficients (additive in the log-transformed model and multiplicative in the original model). Additionally, the apparent difference in coefficient estimates of extreme hazards are only significant in three cases, see Table 5, suggesting that the data transformation may not be necessary. We find that the impact of the other hazard types (i.e., wildfires, tornadoes, ice storms, snow storms and earthquakes) on outward migration is less clearcut and mostly insignificant. This could be because the economic damage from these type of hazards is generally less severe compared to hurricanes, severe storms and floods, and their lower frequency and thus smaller sample size can result in less accurate estimates.
- *Impact on inward migration*: Economic damage from hurricanes, floodings, wildfires, snow storms and earthquakes is associated with lower migration inflows. These findings are consistent in the model that distinguishes between extreme and less severe hazards. However, it is surprising that the significant effects are mostly observed for the less severe events rather than the extreme events (except for hurricanes and wildfires). Again, this could be due to a lower sample size, as there are only a few counties hit by 99th percentile hazards.
- *Indirect economic effects on migration*: We analyzed if there was evidence for an indirect impact of natural hazards on migration through income and unemployment rates. In additional regression models given in the Supplementary Information, the results showed that most hazards had a significant effect on the income and unemployment differential, but the effects were minor. In general, we found that the impact of income and the unemployment rate on migration is small, even though income is one of the key drivers in migration decisions in the literature (Black et al. 2011; Duijndam et al. 2022). However, since this is a macro-level study, the effects of income are probably lost in aggregation (Logan et al. 2016). In other words, while individual differences in income level can play a large role in migration decisions, these differences will be averaged out at the county level. As a result, income appears to only have a small role in driving migration decisions.
- *Impact of distance on migration decisions:* We extended the gravity model to test if migration flows to nearby counties are larger compared to flows to more distant counties, which is a well-established observation in the literature (McLeman and Gemenne 2018). In order to verify this observation, we introduce interaction variables for each type of hazard, which consist of a dummy variable that is activated in the year a hazard occurs and then multiplied by the distance between two counties. For storms and floodings, we indeed find that flows to nearby counties are comparatively larger than to distant counties (at a significance level of 1%). These findings are also supported by the logarithmic interaction model. For hurricanes, there appears to be a greater tendency for people to migrate to more distant counties. However, in the log interaction model, this effect is reversed, suggesting that people relocate to more nearby counties. We believe that this discrepancy between the two models is due to the log transformation, which reduces the influence of extreme events on the estimates. Prior research demonstrated that in the aftermath of Hurricane Katrina, migrants spread all over the country (Fussell et al. 2014). This effect was captured by the original model but not by the logarithmic model, where outliers such as Hurricane Katrina have less influence. To conclude, we note that the coefficients of the interaction variables are small and the R2 reflects that the interaction variables contribute little to the explanatory power of the model.
- *Limitations in the methodology*: In recent years, causality has become an important topic in econometric modelling. While causal inference-based research designs, such as natural control experiments and counterfactuals, have become more popular, this study applies a more conventional regression-based approach. As a result, our statistical framework is limited to showing correlations in the data rather than establishing causal relationships. However, our analysis analyzes the impact of hundreds of hazards on migration, which would be less suitable for natural control experiments and counterfactuals that tend to focus on a single event, see for example, Curtis et al. (2015). Despite this, we aimed to avoid a one-size-fits-all approach by differentiating between types of hazards and taking into account the severity of hazards through their economic damage. In addition, our panel regression methodology provides advantages over other regression approaches since we can control for unobserved factors in the data, thereby reducing the risk of an omitted variable bias and improving the validity of our results. On this note, we assumed that social networks are included in the fixed effects of the model but it would be interesting for future research to specifically account for social networks, as they play a key role in migration destination decisions.
- *Limitations in the data:* Our dataset spans only 12 years, which is relatively short and limits our analysis to estimating the immediate impact of natural hazards on migration, while ignoring the long-term effect. However, as Fussell et al. (2017) shows, the impact of disasters on population growth (a proxy for migration) can vary across different time scales. Another limitation is that the data did not allow us to distinguish between types of migrants. For example, Gutiérrez-Portilla et al. (2018) found different migration responses for natives and foreigners in Spain after an economic shock. Moreover, Pais and Elliott (2006) demonstrated that the migration response to Hurricane Katrina differed along racial and class lines, highlighting the importance of using an intersectional approach to understand how socio-economic and demographic characteristics affect migration patterns. However, due to privacy reasons, these characteristics are not provided in the IRS migration dataset.

To conclude, our results suggest that the effects of various hazards on migration patterns differ significantly. Hurricanes seem to have the most substantial impact, followed by floodings and severe storms, emphasizing the need to distinguish between different types of hazards. Furthermore, our study confirms prior research, which suggests that people tend to move to nearby areas in the event of a hazard. Even though the estimated effect is small, this behavior may be concerning, as it implies that individuals could still be at a high risk of experiencing future disasters. These findings offer a promising direction for future research, as it highlights the importance of investigating why people tend to relocate to nearby areas during natural hazards, and what the potential risks of this behavior may be.

## Supplementary Information

Below is the link to the electronic supplementary material.

Supplementary file1 (DOCX 19 kb)

## Data Availability

The data sets used in this study are publicly available.

## Footnotes

1 In the SHELDUS database we find that many counties report the exact same hazard damage after an event, which is not realistic.

2 $$\left({e}^{-0.1037*1}-1\right)*100\%=-9.85\%$$ and $$\left({e}^{-0.1411*1}-1\right)*100\%=-13.16\%$$.

3 $\left({e}^{\left(0.0552*1\right)+(-0.0195*\text{distance})}-1\right)*100\%$, where distance is measured in 1000 s miles.

4 For the logarithmic model, we added + 1 to all hazard damages to avoid taking a log of zero. Hence, an increase of 1000 USD corresponds to an increase of $\frac{1001-1}{1}*100\%=\text{100,000}\%$.

## Code availability

The code and data sets used in this study are stored in an open access repository.
