# Supplementary material for: Economic damage from natural hazards and internal migration in the United States
Source: Nat Hazards (Dordr). 2024 Nov 6;121(4):4985–5005. doi: 10.1007/s11069-024-06987-2 (PMC11968479; doi:10.1007/s11069-024-06987-2)
Supplement: Supplementary file 1 — Supplementary file1 (DOCX 19 kb) [file 11069_2024_6987_MOESM1_ESM.docx]

Supplementary Information - Economic damage from natural hazards and internal migration in the United States

Table S1. Impact of natural hazards on income and unemployment

|  | **Fixed effects model income** | **Fixed effects model unemployment** |
| --- | --- | --- |
| O_hurricane | 0.0111***  (0.0003) | -0.1532***  (0.0009) |
| D_hurricane | -0.0100***  (0.0004) | 0.0201***  (0.0015) |
| O_storm | -0.0031***  (0.0010) | 0.0138***  (0.0026) |
| D_storm | 0.0002  (0.0010) | -0.0078***  (0.0024) |
| O_flood | 0.0277***  (0.0044) | 0.0001  (0.0082) |
| D_flood | -0.0221***  (0.0037) | 0.0087  (0.0086) |
| O_tornado | 0.0184***  (0.0066) | -0.0063  (0.0178) |
| D_tornado | -0.0098  (0.0075) | -0.0328  (0.0248) |
| O_fire | -0.0191***  (0.0008) | -0.0418***  (0.0084) |
| D_fire | 0.0189***  (0.0007) | 0.0420***  (0.0022) |
| O_snow | -0.7563***  (0.0474) | 0.3336**  (0.1467) |
| D_snow | 0.7873***  (0.0504) | 0.4573***  (0.1533) |
| O_icestorm | -0.0044  (0.0064) | -0.1675***  (0.0184) |
| D_icestorm | -0.0050  (0.0067) | 0.2422***  (0.0211) |
| O_earthquake | -0.1206***  (0.0164) | 0.3642***  (0.0604) |
| D_earthquake | 0.1170***  (0.0179) | -0.2119***  (0.0727) |
| R2 Overall | 0.0028 | 0.0009 |

Table S2. Income-corrected model

|  | **Income-corrected model** | **Original model (standardized*)** |
| --- | --- | --- |
| Income differential | 0.0888***  (0.0133) | 0.0873***  (0.0127) |
| Unemployment differential | -0.1107***  (0.0046) | -0.0896***  (0.0040) |
| O_hurricane | 2.5968***  (0.1858) | 2.5309***  (0.1885) |
| D_hurricane | -2.5584***  (0.3514) | -2.8157***  (0.4038) |
| O_storm | 0.1994***  (0.0679) | 0.2182***  (0.0672) |
| D_storm | 0.0532  (0.0428) | 0.0551  (0.0437) |
| O_flood | 0.1900***  (0.0591) | 0.1810***  (0.0603) |
| D_flood | -0.2994***  (0.0994) | -0.3542***  (0.1008) |
| O_tornado | 0.0894  (0.0898) | 0.1204  (0.0906) |
| D_tornado | 0.0092  (0.1674) | -0.0088  (0.1649) |
| O_fire | -0.0307  (0.0187) | -0.0196  (0.0196) |
| D_fire | -0.1456***  (0.0221) | -0.1402***  (0.0217) |
| O_snow | -0.0082  (0.0385) | 0.0761*  (0.0438) |
| D_snow | -0.0605**  (0.0385) | -0.1094**  (0.0453) |
| O_icestorm | 0.0243  (0.0466) | 0.0398  (0.0512) |
| D_icestorm | -0.0430**  (0.0432) | -0.0911  (0.0554) |
| O_earthquake | 0.0880  (0.2424) | 0.0492  (0.0581) |
| D_earthquake | -0.1793**  (0.0738) | -0.1925**  (0.0805) |
| Pseudo R2 | 0.4654 | 0.4666 |

*We standardized the economic damage by dividing each hazard’s economic damage by the maximum damage of the respective hazard type. Hence, the maximum damage of each hazard type is 1, for both the original model and the income-corrected model, to compare the estimated coefficients.
